# Supplementary material for: A Combined Metabolome and Transcriptome Reveals the Lignin Metabolic Pathway during the Developmental Stages of Peel Coloration in the ‘Xinyu’ Pear
Source: Int J Mol Sci. 2024 Jul 8;25(13):7481. doi: 10.3390/ijms25137481 (PMC11242026; doi:10.3390/ijms25137481)
Supplement: Supplementary file 1 [file ijms-25-07481-s001.zip › Supplementary Materials 20240630/Figure S1.docx]

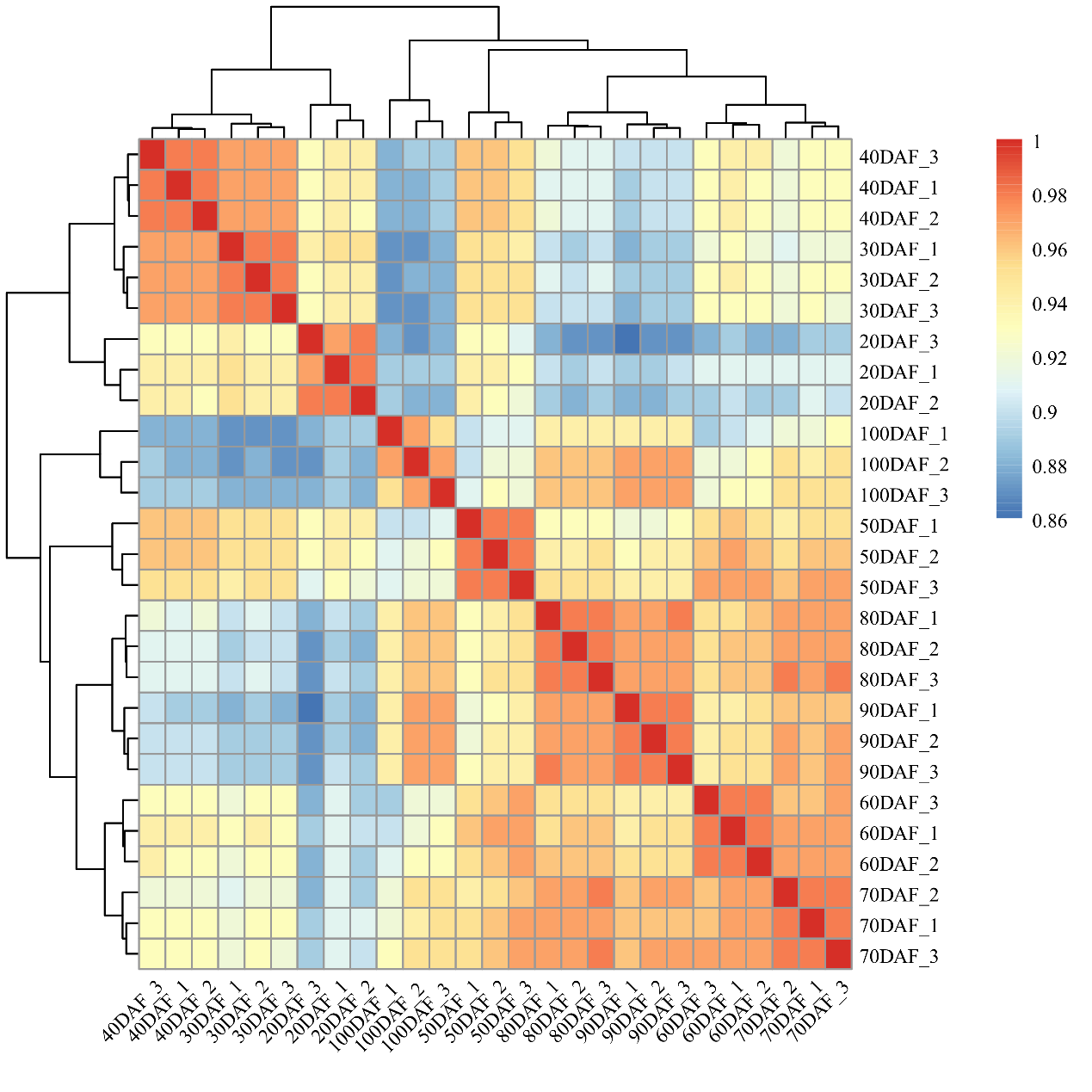


**Figure S1** **Correlation analysis of expression levels of identified genes by transcriptomic in ‘Xinyu’ peel during 9 different development stages.** The correlation between samples is calculated using the cor function in R. The color scale on the right indicates the Spearman correlation of samples from 0.86 (blue) to 1 (red).
